# Numerical simulation of crack propagation and AE precursor characteristics in coal failure under confining pressures

**DOI:** 10.1038/s41598-025-08281-y

**Published:** 2025-07-02

**Authors:** Gang Jing, Shuai Wang, Leilei Zhao, Fa Dong, Yi Fan

**Affiliations:** 1 Key Laboratory of Xinjiang Coal Resources Green Mining, Xinjiang Institute of Engineering, Ministry of Education, Ürümqi, 830023 China; 2 Xinjiang Key Laboratory of Coal-bearing Resources Exploration and Exploitation, Xinjiang Institute of Engineering, Ürümqi, 830023 China; 3 Xinjiang Engineering Research Center of Green Intelligent Coal Mining, Xinjiang Institute of Engineering, Ürümqi, 830023 China; 4 School of Mining Engineering and Geology, Xinjiang Institute of Engineering, Ürümqi, 830023 China

**Keywords:** Coal burst, Acoustic emission, Crack propagation, Confining pressure, Precursor indicator, Civil engineering, Natural hazards

## Abstract

Predicting coal bursts in deep mining requires understanding the crack propagation and acoustic emission (AE) characteristics of coal under triaxial stress conditions. This study employs discrete element simulation to investigate the mechanical behavior and AE responses of cylindrical coal specimens under varying confining pressures. The results demonstrate that peak strength increases with loading rate, particularly under low confining pressures, while higher pressures dampen this sensitivity. Volumetric strain-stress curves effectively identify crack initiation and damage thresholds, which increase with confining pressure while reducing the duration of unstable crack propagation. The proportion of tensile cracks gradually decreases while the proportions of shear and mixed-mode cracks increase. Specimens remain stable when *β*_*t*_-value < 1 and enter instability when *β*_*t*_-value > 1, with *β*_*t*_-value remaining unaffected by confining pressure variations., Meanwhile, *b*-value exhibit a “sharp-drop-plateau” pattern prior to failure. These findings provide a theoretical framework for interpreting AE signals in deep mining environments, enhancing coal burst prediction capabilities through mechanistic insights into confining pressure effects on crack evolution and precursor indicator.

## Introduction

Coal burst refers to a dynamic disaster phenomenon in coal mines where coal-rock masses under high-stress conditions undergo sudden failure, releasing enormous energy that causes rock fracture, ejection, vibration, and even explosion^1^. Characterized by abrupt occurrence, concentrated energy release, and extensive damage, coal bursts have long challenged the mining engineering field in terms of prevention and control^2^. Coal burst is closely associated with the progressive evolution of internal crack structures in coal-rock masses, involving microscopic processes such as crack initiation, propagation, and coalescence^3^. However, the weak and complex precursor signals of coal burst, combined with the three-dimensional stress environments of coal mining and the inherent heterogeneity and nonlinear mechanical behavior of coal-rock materials, render existing monitoring technologies inadequate for precise early warning^4^. Notably, significant variations in crack propagation patterns and energy release mechanisms under different confining pressure conditions undermine the reliability of traditional warning index systems based on uniaxial/biaxial tests in deep mines with complex stress environments. Therefore, systematically investigating the influence of confining pressure variations on the evolutionary characteristics of acoustic emission precursor indicators in coal-rock masses holds critical scientific significance and engineering value for overcoming bottlenecks in coal burst prevention and control.

Studies show that coal bursts release immense energy alongside physical signals like acoustic emission^5^ (AE), Charge induction^6^, and electromagnetic emission^7^. AE technology, with its real-time, continuous, and highly sensitive capabilities, has become a key tool for monitoring coal-rock failure processes^8^. Researchers globally have conducted extensive research on the correlation between AE parameters and coal failure. For instance, Zhao et al.^9^ employed AE technology to conduct a quantitative analysis of the coal burst process, classified the signals into five categories based on variations in AE energy, and proposed early-warning indicator. Khadivi et al.^10^ studied the basic principles of blasting and brittleness, proposed a new blasting-brittle ratio, and used high-speed imaging and AE techniques to investigate crack evolution and specimen failure behavior, thereby validating the effectiveness of the proposed ratio. Yang et al.^11^ analyzed the correlation dimension and Hurst exponent of fractal characteristics and found that variations in the Hurst exponent during sudden rock instability hold guiding significance for detecting pre-failure precursors. Li et al.^12^ investigated the fractal characteristics of rocks at various loading stages and found that the fractal dimension in the final stage consistently remained lower than those in preceding stages, enabling the identification of impending instability. Kong et al.^13^ studied AE variations under true triaxial loading conditions and proposed that the correlation dimension can reflect internal damage levels in rock strata, with its gradual decrease serving as a precursor to rock instability. Liu et al.^14^ employed true triaxial and AE equipment to investigate the effects of different bedding angles on rockburst in sandstone specimens. Based on the multifractal theory, Long et al.^15^ studied the damage evolution law and multifractal parameter changes of gas bearing coal, revealing the precursor information of gas bearing coal seam damage. The results indicate that cracks within the coal continue to accumulate and propagate before the failure of gas bearing coal. Ma et al.^16^ employed discrete wavelet analysis to conduct an in-depth study of AE signals and proposed that the time of first occurrence of negative α value in the Lipschitz exponent could serve as a failure prediction time. Sun et al.^17^ designed a self-organizing mapping neural network array to extract AE precursor waveforms for rockburst and brittle failure. Khadivi et al.^18^−^19^ employed AE and high-speed imaging techniques to investigate the relationship between macro-crack and micro-crack propagation in sandstone, coal, and granite under Brazilian splitting.

In recent years, numerous scholars have combined the “AE *b*-value” with AE technology to analyze damage and fracture instability in solid materials. For example, Huang et al.^20^ investigated failure precursor characteristics of weathered granite by synchronously analyzing critical slowing-down theory and b-values, enabling accurate identification of rock failure patterns. Chen et al.^21^ conducted laboratory direct shear tests and AE experiments on standard cylindrical specimens with different rock surface roughness levels. The evolution of AE *b*-value indicated that the macroscopic fracture surfaces of bonded interfaces resulted from repeated transitions between microcracks of varying sizes. Colombo et al.^22^ analyzed correlations between AE *b*-value and loading characteristics, damage evolution parameters, and crack morphology during concrete fracture, demonstrating that dynamic *b*-value evolution precisely characterizes the fracture mechanics behavior of crack nucleation-propagation-coalescence in concrete. Xiao et al.^23^ performed AE monitoring during coal sample failure and examined variations in stress release rate, AE counts, AE energy, and *b*-value during crack propagation.

Although existing studies have made significant advances in AE monitoring of precursor characteristics, a systematic understanding of the regulatory mechanisms governing AE precursor evolution under varying confining pressures in triaxial stress states remains lacking. Notably, under high confining pressure constraints, coal-rock crack propagation transitions from tensile-dominated to shear-dominated modes, leading to pronounced changes in AE precursor evolutionary characteristics. However, the quantitative relationship between these evolutionary patterns and confining pressure magnitudes remains unclear.

Based on the above research gaps, this study hypothesizes that confining pressure modulates the frequency-energy distribution of acoustic emission (AE) signals by altering the proportion of crack types, specifically that higher confining pressures suppress the dominance of tensile cracks and promote the propagation of shear/mixed-mode cracks, leading to lower AE signal frequency and higher energy release, and that this transition in crack propagation modes can be quantitatively characterized by AE parameters.

This study systematically investigates the crack propagation laws and evolutionary characteristics of AE precursor indicators in coal-rock specimens under varying confining pressures using the Particle Flow Code method (PFC). By integrating moment tensor analysis with time-series evaluation of AE parameters, this research aims to reveal the regulatory mechanisms of confining pressure on crack propagation modes and AE signals, establish a theoretical framework for AE-based early warning of coal instability in deep mines, and provide a scientific basis for precise prediction of coal burst disasters.

## Modeling procedures

### Basic principle of grain-based model

PFC (Particle Flow Code), a numerical simulation software meticulously developed by Itasca Consulting Group, falls within the category of discrete element models^24^. It employs rigid spherical bodies to simulate particles within rock masses, where particle motion strictly adheres to Newton’s laws of mechanics. The simulation fundamentally initiates when the model deviates from static equilibrium conditions. In PFC, the cementation state of minerals is represented by implementing contact models between particles, with the rupture of contacts being interpreted as microcrack generation. Through the interactions between particles, the macroscopic mechanical properties of the model can be thoroughly investigated. Given the complexity of three-dimensional models and the extended computation time required for simulations, this study employs a two-dimensional model (simplifying cylinders in the two-dimensional system as rectangular blocks). The particle flow software version utilized in this research is PFC^2D^ 5.00.25.

**Fig. 1:**
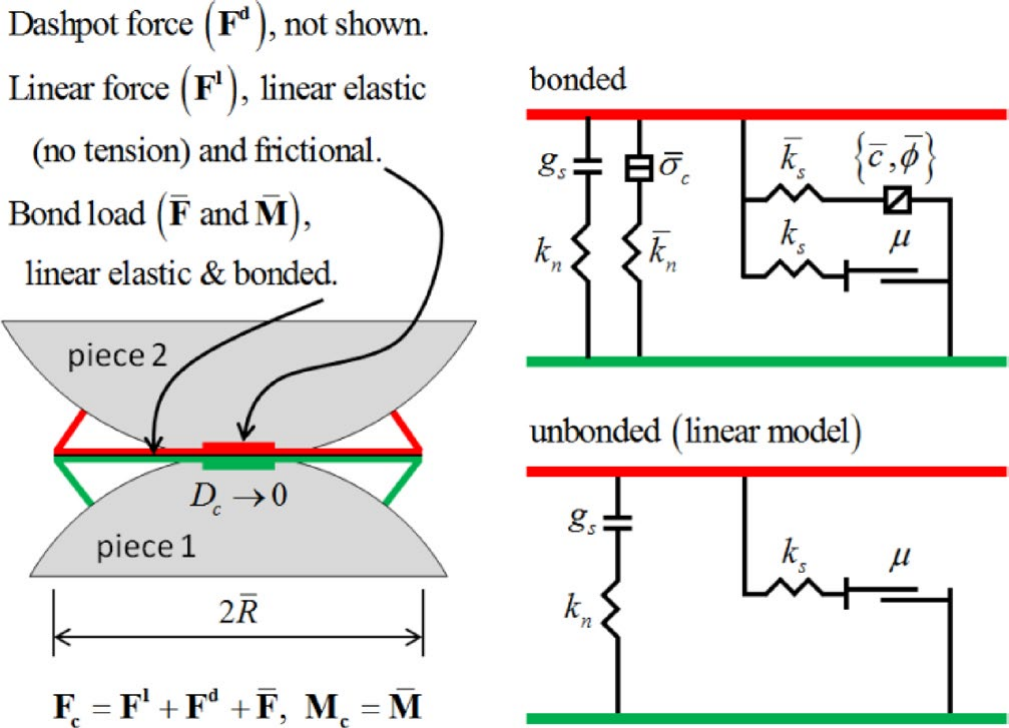
Linear parallel bond models for behavior and rheological components^26^.

The mechanical behavior of bonded materials is characterized using linear parallel bonds and their rheological components, as illustrated in Fig. 1. The parallel bond model provides two interfacial behaviors: (1) An infinitesimal linear elastic (tension-free) frictional interface that carries force; (2) A finite-sized linear elastic bonded interface that carries both force and moment. The first interface is equivalent to a linear model: it does not resist relative rotation and regulates slip by imposing a Coulomb limit on shear forces. The second interface is termed a “parallel bond” because it acts in parallel with the first interface when bonded. When the second interface is bonded, it resists relative rotation with linear elastic behavior until strength limits are exceeded and the bond breaks. When the second interface is unbonded, it does not carry loads. The unbonded linear parallel bond model is equivalent to the linear model.

A parallel bond can be conceptualized as a set of elastic springs with constant normal and shear stiffnesses, uniformly distributed over a two-dimensional rectangular area (in 2D) or a three-dimensional circular cross-section centered at the contact point (in 3D). These springs act in parallel with the springs of the linear component. After parallel bond formation, relative motion at the contact generates forces and moments within the bonded material. These forces and moments act on the two contacting components and relate to the maximum normal and shear stresses developed at the bond perimeter in the bonded material. If any of these maximum stresses exceed their respective bond strengths, the parallel bond fractures, and the bonded material—along with its associated forces, moments, and stiffness is removed from the model^25^.

The parallel bond can be described by a shear force and a normal spring connecting the two particles. Both moments and forces can be transmitted through the bond. The maximum tensile and shear stresses acting on the bond are given by:

1 $$\:\left\{\begin{array}{l}{\sigma\:}_{t\:max}=\frac{{\bar{F}}_{n}}{A}+\frac{\left|\stackrel{-}{M}\right|}{l}\stackrel{-}{R}\\\:{\tau\:}_{max}=\frac{{\stackrel{-}{F}}_{s}}{A}\end{array}\right.$$.

In the equations, *σ*_*tmax*_ and *τ*_*max*_ represent the maximum normal stress and maximum shear stress, respectively; *F*_*n*_ and *F*_*s*_ denote the normal and tangential components of the parallel bond; *M* is the moment of the parallel bond; *A* and *I* are the cross-sectional area and moment of inertia of the bond; *R* is the bond radius. If the tensile stress exceeds *σ*_*t max*_, tensile failure occurs. If the shear stress exceeds *τ*_*max*_, shear failure occurs, and the bond degenerates into a linear model when it fractures.

### Moment tensor modeling principle for acoustic emission

In PFC^2D^ discrete element simulations, the traditional approach treats individual contact fractures as independent AE events. However, this method has significant limitations: all events are assigned identical fracture strengths, which assumes identical fracture strengths for all events and neglects the spatiotemporal correlation of microcracking. This leads to significant discrepancies with laboratory AE monitoring results, where multiple microcracks often coalesce within localized zones^27^. To improve simulation accuracy, this study employs a criterion defining microcrack clusters generated at the same spatial location within a similar time window as a single AE event. By reflecting the spatiotemporal correlation of the fracture process, this criterion significantly improves the physical authenticity of AE event characteristics.

Moment tensor theory, an important analytical tool in rock mechanics and mining engineering, can quantitatively characterize source mechanisms and their spatiotemporal evolution laws. Originating in seismology, this theory reveals rock mass failure mechanisms through inversion of dynamic wavefield data from seismic sources. This study extends its application to source analysis of underground coal mine dynamic disasters and establishes a moment tensor calculation method within the PFC^2D^ discrete element framework. In the PFC^2D^ discrete element model, particle displacement fields and contact force fields are known quantities. When a bond fractures under external loading, the particles at both ends of the microcrack are defined as source particles. Based on discrete element contact mechanics, the moment tensor *M* can be calculated using Eq. (2):

2 $$\:{M}_{ij}=\sum\:\varDelta\:{F}_{i}{\delta\:}_{j}$$.

Where, *S* represents the fracture surface; *F*_*i*_ represents the *i*^th^ unbalanced contact force; *δ*_*j*_ represents the distance between the center of the microcrack and the contact point. In a two-dimensional space, the moment magnitude of acoustic emission can be calculated according to the following formula^28^:

3 $$\:M=\left(\frac{2}{3}lg{M}_{0}-6\right)$$.

To distinguish crack propagation modes, tensor decomposition of the moment tensor is required. This study adopts the improved fracture classification criterion proposed by Liu et al.^29^ Based on the isotropic component method (ISO) proposed by Ohtsu^30^, the following classification criteria are established:

4 $$\:R=\frac{{tr\left(M\right)}^{*}100\%}{\left|tr\left(M\right)+\sum\:{{m}_{i}}^{*}\right|}$$.

**Fig. 2:**
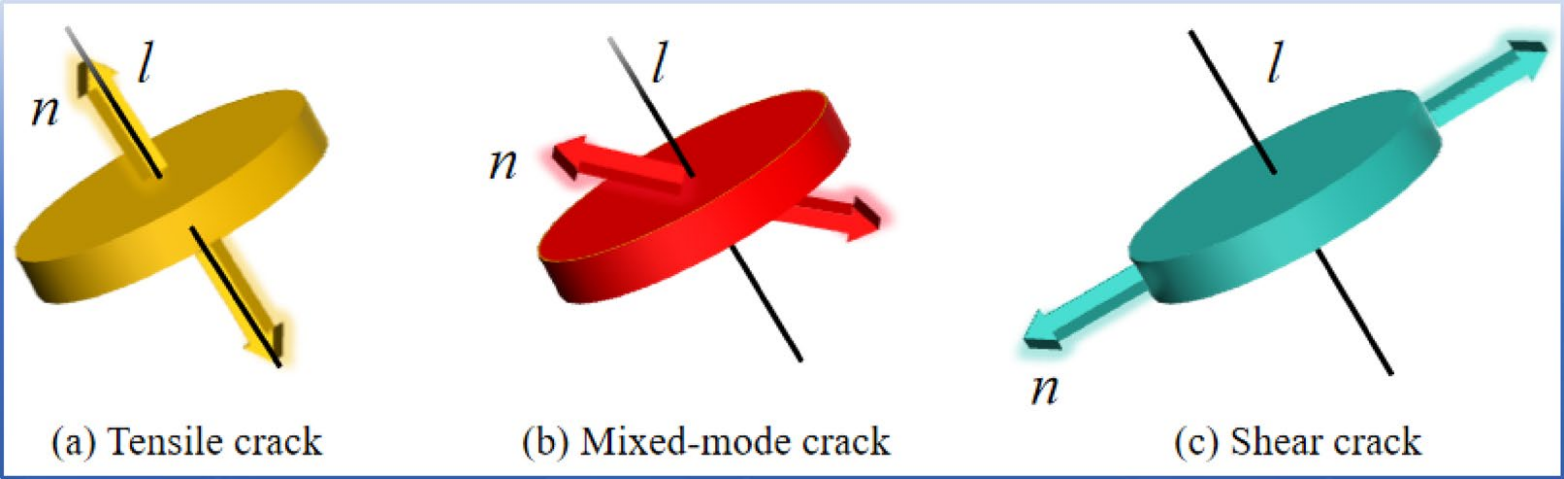
Different crack failure modes.

The value range of the isotropic ratio *R* is from-100% (representing pure implosion) to 100% (representing pure tension), and *R* = 0 represents pure shear. AE events are classified as follows: implosion events when *R*<-30 shear events when-30 < *R* < 30, and tension events when *R* > 30^24^. By simulating AE signals through moment tensor analysis, the changing characteristics of crack types during the instability and failure of coal at different loading stages can be obtained. Cracks can be classified, and the evolution and propagation laws of different types of cracks can be analyzed, as shown in Fig. 2.

## Modeling of rectangular coal sample by Grain-Based model

**Fig. 3:**
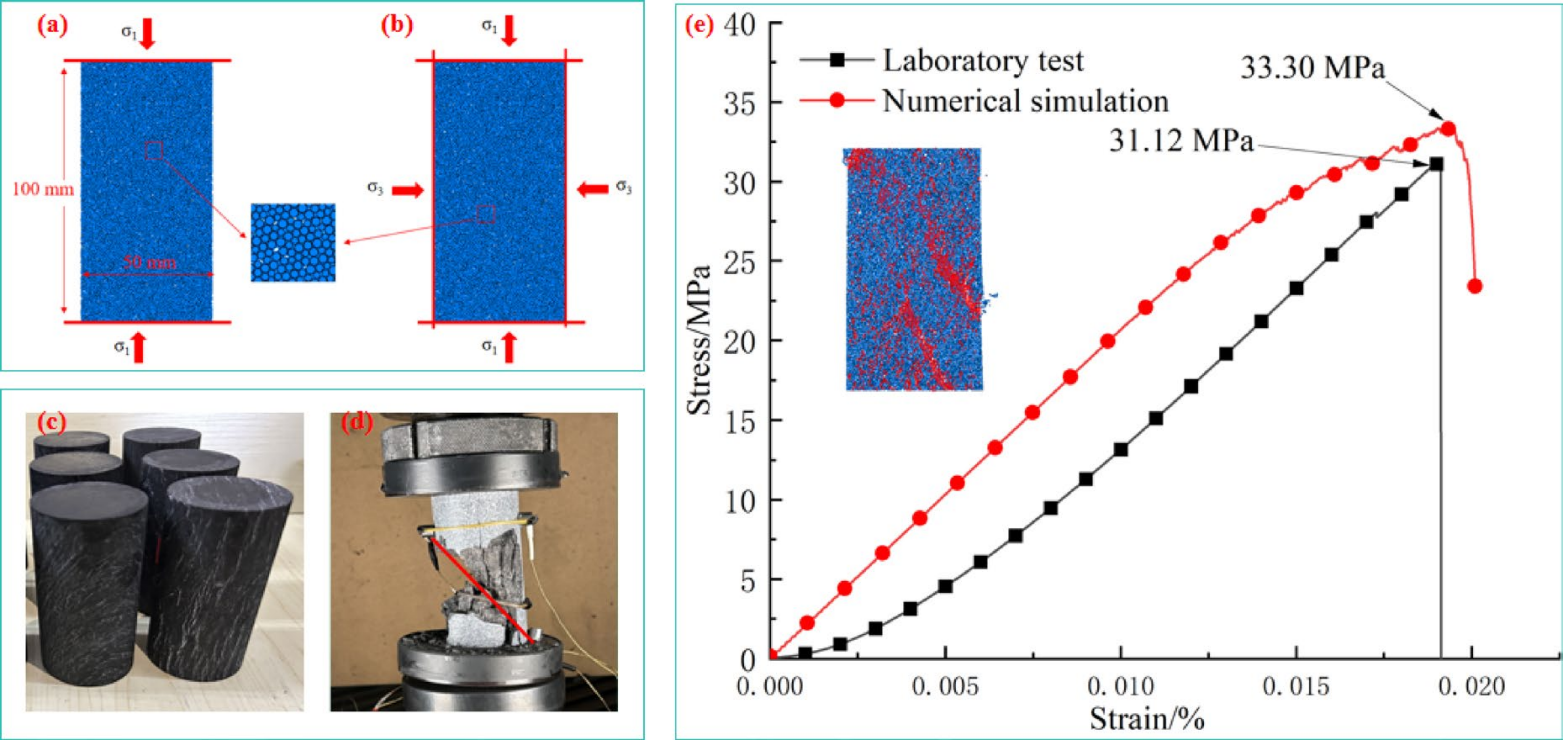
Comparison of numerical simulation and experimental test results.

The model dimensions established via numerical simulation were consistent with the samples used in the actual laboratory, resulting in a diameter of 50 mm and a length of 100 mm. The particle size range was set at 0.4–0.66 mm, with the ratio of the minimum to maximum particle size being 1.66^31^. Considering both computational capacity and efficiency, two-dimensional models were adopted for both uniaxial and triaxial compression tests, as shown in Fig. 3(a). During testing, particles at the top and bottom moved relative to each other at a constant velocity v to simulate the sample compression process. The numerical model for simulating triaxial compression is illustrated in Fig. 3(b). After model construction, stresses were applied simultaneously in both the axial and lateral directions to reach preset stress levels, mimicking the triaxial stress state. Once the model achieved equilibrium, constant velocities *v* were applied to the top and bottom of the model respectively to simulate the sample compression process under laboratory triaxial conditions.

The approach of the numerical simulation in this paper involves calibrating numerical parameters based on the macroscopic parameters of uniaxial compression tests (coal sample Fig. 3(c) and uniaxial loading Fig. 3(d)) to achieve good consistency between simulated and experimental stress-strain curves of uniaxial compression. Subsequent application of lateral constraints was then performed to simulate the mechanical properties and acoustic emission characteristics of specimens under triaxial loading conditions, aiming to investigate crack propagation features under triaxial stress states.

In PFC^2D^ numerical simulations, parameter calibration is an indispensable step. The selection of mesoscopic parameters directly determines the reliability of simulation results. However, due to the complexity of mesoscopic parameter calibration, it remains challenging to directly determine the micromechanical parameters of simulated particles. In engineering practice, the trial-and-error approach is a commonly used method for parameter calibration. This method involves iteratively adjusting mesoscopic parameters through comparative analysis between laboratory macroscopic mechanical parameters and numerical simulation results until a high degree of consistency is achieved. The calibration process prioritized matching the macro-mechanical properties of coal samples through a stepwise adjustment of mesoscopic parameters. First, the elastic modulus and Poisson’s ratio were calibrated by fitting the linear elastic stage of the uniaxial compression stress-strain curve. This involved adjusting particle stiffness and bond modulus until the simulated elastic modulus closely matched the experimental value. Next, strength-related parameters were calibrated to reproduce the peak strength and post-peak behavior. By iteratively tuning the parallel bond strength and particle friction coefficient, the simulated uniaxial compressive strength was aligned with the laboratory result. This sequential calibration ensured that both elastic deformation and failure characteristics of the numerical model matched the experimental data.

The mesoscopic parameters adopted in this study are listed in Table 1, which were determined based on experimental data from Hongqinghe coal samples and validated through multiple rounds of numerical parameter tuning.

The numerical simulation experiment was conducted using the Hongqinghe coal sample as a reference, with a stress-strain curve obtained at a loading rate of 0.3 mm/min. The uniaxial compressive strength and elastic modulus of the coal sample were 31.2 MPa and 1.77 GPa, respectively. The comparison between numerical simulation and experimental results is shown in Fig. 3(e). After calibration, the model achieved an uniaxial compressive strength of 32.16 MPa and an elastic modulus of 2.06 GPa. The macroscopic mechanical parameters of the numerical model are in close agreement with the measured parameters of the target coal sample, as detailed in Table 2.

As shown in Fig. 3(e), although the stress-strain curves from this study’s numerical simulation exhibit good correspondence with laboratory results, the numerical simulation does not capture the compaction stage. In laboratory tests, most coal samples display concave-upward curves throughout the loading process, making it difficult to observe a distinct linear elastic stage. This is attributed to the low strength and high brittleness of coal samples, where the simultaneous generation of elastic energy and dissipated energy obscures the elastic phase.

The post-peak stress drop in the experiment originates from the rapid penetration of natural fractures in coal, while the PFC^2D^ model uses rigid particles and cannot fully reproduce the instantaneous failure of the micro-fracture network. However, by setting the frictional sliding mechanism after bond failure, the model captures the post-peak strain softening trend, and the volumetric strain reversal point is consistent with the experimental law, verifying the model’s capability to characterize the crack propagation stage.

The PFC^2D^ model employs rigid particles to simulate specimen deformation. However, the compaction stage of coal-rock materials cannot be replicated by the extrusion-collision interactions between rigid spheres, leading to challenges in simulating the stress-strain behavior during this phase. Nevertheless, the numerical simulation effectively captures the strain softening stage during the unstable crack propagation phase.

**Table 1:** Simulate the mesoscopic parameters of laboratory tests.

Basic parameters of particles	Value	Parallel bond parameters	Value
*R*_min_ (mm)	0.3	*E* (GPa)	1.62
*R*_max_ (mm)	0.5	*k*_*n*_ */k*_*s*_	10
(kg/m^3^)	1650	*σ*_*t*_ (MPa)	20.2
*n*/%	0.1	*c* (MPa)	20.2
Particle friction coefficient	0.3	*φ*	20
Particle stiffness ratio	0.5	-	-

**Table 2:** Comparison of numerical simulation and experimental mechanical properties.

Mechanical parameters	Laboratory test	Numerical simulation
*σ*_*c*_ (MPa)	31.12	33.30
*E* (GPa)	1.77	2.06
*ε*_*m*_ (%)	0.018	0.020
*ε*_*c*_ (%)	0.018	0.019

## Numerical simulation results

### Temporal and Spatial evolution characteristics of AE

**Fig. 4:**
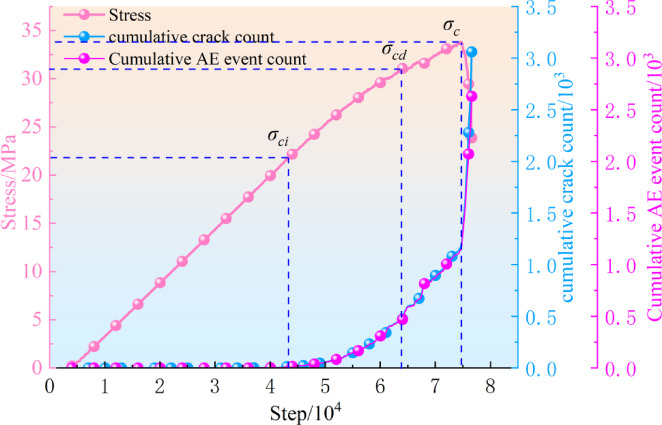
The relationship between the number of crack and AE events is simulated.

The stress curve, cumulative crack count, and cumulative AE event count from the numerical simulation are presented in Fig. 4. The curves of cumulative AE events and cumulative cracks overlap because each individual crack is treated as a single AE event in this simulation. According to the trend of the cumulative AE event curve, almost no AE signals are generated during the initial loading phase. Therefore, based on the characteristics of the simulated AE curve, the numerical loading process can be divided into four stages: ①Linear Phase: During this phase, particles are fully compressed against each other. Due to the rigid nature of the particles, no compaction stage occurs. ②Elastic Phase (Stable Crack Propagation Phase): Strain softening appears, and stable AE signals are generated. ③Plastic Phase (Unstable Fracture Propagation Phase): AE events increase rapidly, and specimen failure accelerates until reaching the peak stress. In the figure, *σ*_*ci*_ and *σ*_*cd*_ represent the crack initiation stress and crack propagation stress threshold, respectively. ④Post-Peak Phase: After the peak stress *σ*_*c*_, the specimen becomes unstable, and the number of AE events reaches its maximum.

**Fig. 5:**
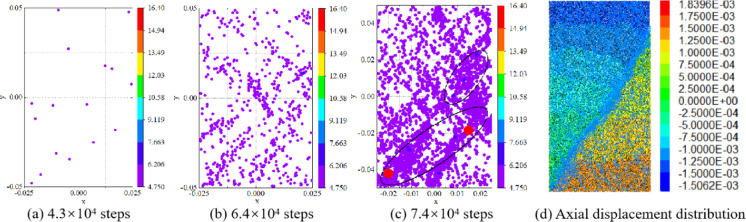
AE spatial orientation.

The spatial localization of cracks during loading is presented in Fig. 5. In the PFC model, crack propagation locations and energy releases can be directly recorded, enabling visualization of crack distributions at different loading steps. The coordinates of solid circles in the figure denote crack positions within the specimen, with color intensity indicating energy release magnitudes.

Crack localization maps at 4.3 × 10^4^, 6.4 × 10^4^, and 7.4 × 10^4^ loading steps are illustrated in Fig. 5(a), (b), and (c), respectively. At 4.3 × 10^4^ steps (prior to the crack initiation stress threshold *σ*_*ci*_), only 16 AE events were detected, indicating the crack incubation phase. As the specimen entered the stable crack propagation stage (6.4 × 10⁴ steps), AE event counts increased significantly, with cracks accumulating locally and forming dense clusters. The low energy releases during these two stages suggest dominant microcrack initiation. At 7.4 × 10⁴ steps (unstable crack propagation phase), AE event counts peaked as microcracks coalesced to form a macroscopic failure surface.

Figure 5(c) reveals two primary crack zones at failure, which correlate well with the axial displacement distribution shown in Fig. 5(d). These results validate the consistency between the numerical model developed in this study and laboratory test observations.

### Mechanical properties of coal under different confining pressures

As demonstrated by the above analysis, the numerical model developed in this study exhibits excellent agreement with laboratory tests, validating the rationality of the designed simulation parameters. Building upon the mesoscopic parameters calibrated for uniaxial compression, this work conducts numerical triaxial compression tests with confining pressures of 5 MPa, 15 MPa, 25 MPa, 35 MPa, 45 MPa, and 55 MPa, and loading rates of 0.06, 0.3, and 0.6 mm/min. The objectives are to investigate the strength characteristics, deformation behavior, and crack propagation features of specimens under different confining pressures. Through moment tensor analysis of AE signals, the precursor characteristics of specimen instability during triaxial compression are systematically explored.

**Fig. 6:**
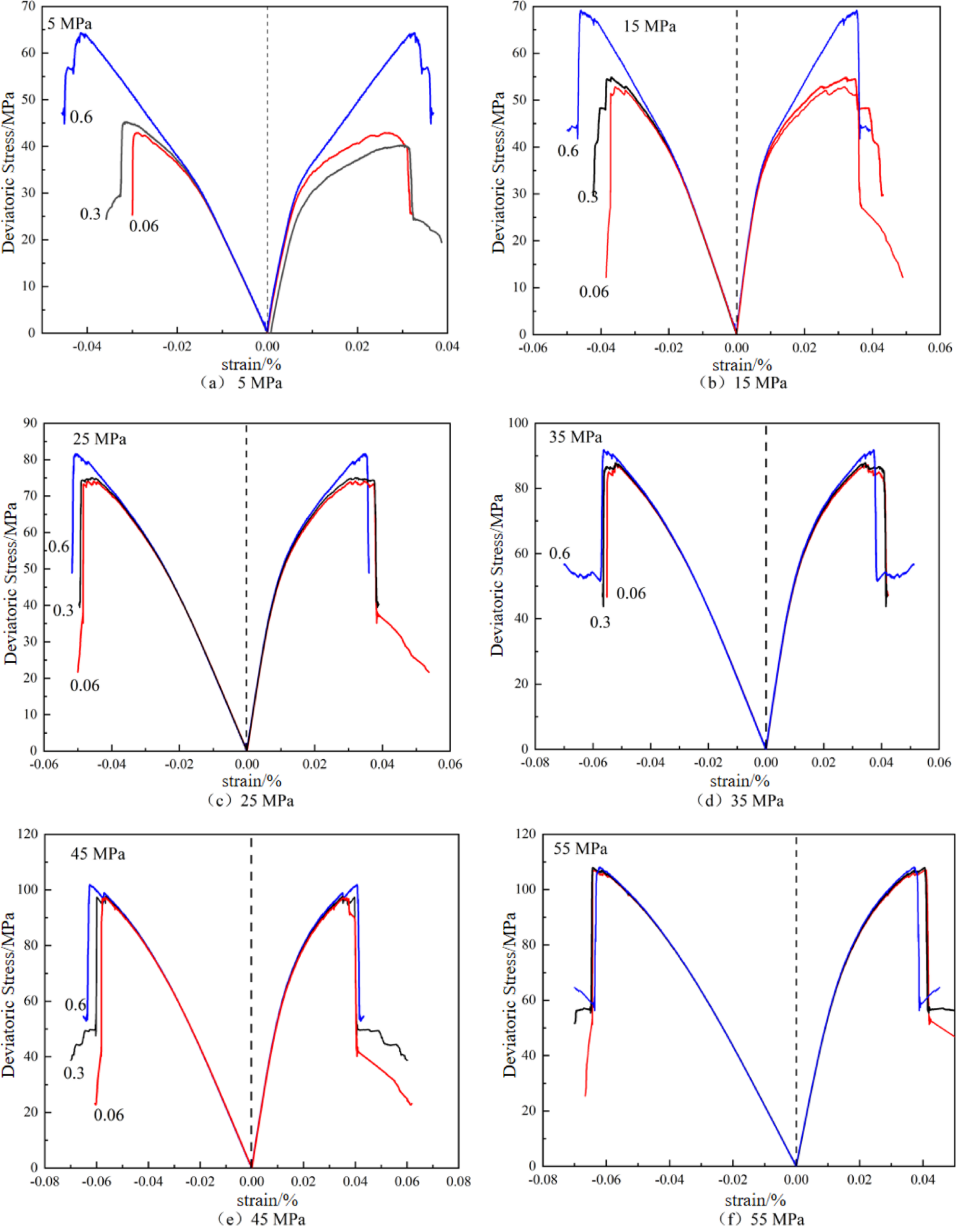
Stress-strain curves of numerical samples with different strain rates under different confining pressures.

The stress-strain curves of numerical specimens under different confining pressures and loading rates are presented in Fig. 6. Stress-strain curves under different conditions exhibit similar trends. Similar to the uniaxial compression simulations described earlier, numerical specimens do not display a compaction stage. This is because PFC^2D^ employs rigid spherical particles, which only generate interparticle forces during compression. In contrast, laboratory coal samples contain abundant primary cracks and pores. During initial compression, these pores and cracks gradually close, resulting in strain hardening and concave-upward curves in stress-strain plots. This discrepancy highlights the differences between numerical and laboratory simulations. Nevertheless, all curves exhibit a linear elastic stage (stable crack propagation), plastic stage (unstable crack propagation), and post-peak stage, confirming the good consistency between the numerical model and experimental results.

Notably, stress-strain curves show distinct differences under varying loading rates. Peak strength increases with higher loading rates. The variation of peak strength with loading rates differs significantly across different confining pressures.

**Fig. 7:**
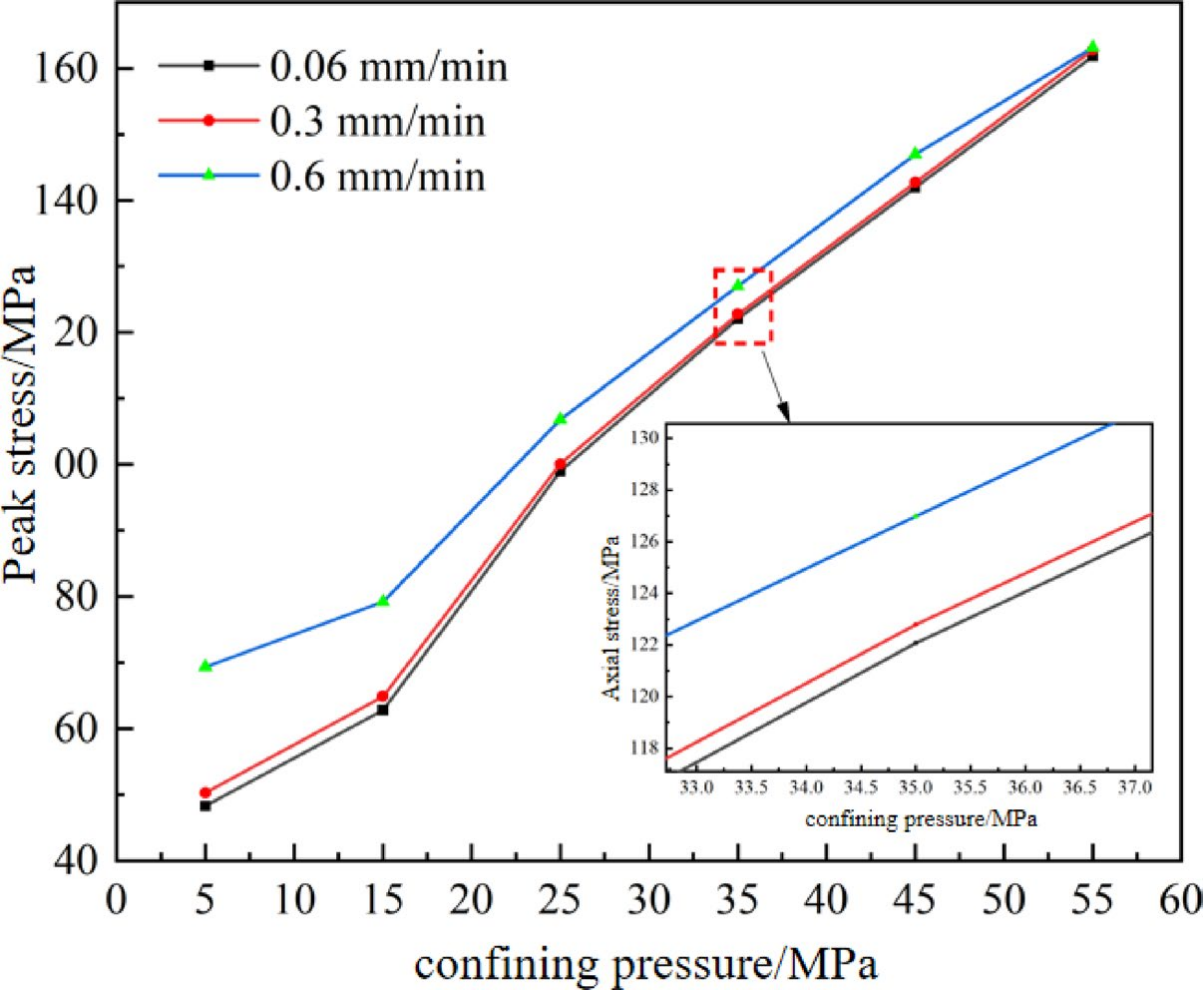
Variation trend of peak strength under different confining pressures.

The variation trend of peak strength under different confining pressures is shown in Fig. 7. Overall, peak stress increases with higher loading rates. As confining pressure increases, peak strength exhibits a linear growth pattern. At a confining pressure of 5 MPa, peak strengths at loading rates of 0.06, 0.3, and 0.6 mm/min are 48.29 MPa, 50.26 MPa, and 69.35 MPa respectively, corresponding to increases of 1.97 MPa and 19.09 MPa. At 55 MPa confining pressure, peak strengths under the three loading rates are 161.79 MPa, 162.74 MPa, and 163.13 MPa, with increments of 0.95 MPa and 0.39 MPa. Compared to high confining pressures, the effect of loading rate on peak strength is more significant at low confining pressures, indicating greater sensitivity to loading rate. Under high confining pressures, peak strength shows minimal variation with increasing loading rates.

### Evolution characteristics of coal crack propagation under different confining pressures

This section investigates the AE response patterns and crack propagation mechanisms of coal under different confining pressures using numerical specimens with a loading rate of 0.3 mm/min. Based on the AE principles of numerical simulation, cracks are classified into tensile cracks, shear cracks, and mixed cracks (implosion type) using the isotropy ratio R.

#### Distribution characteristics of crack types

**Fig. 8:**
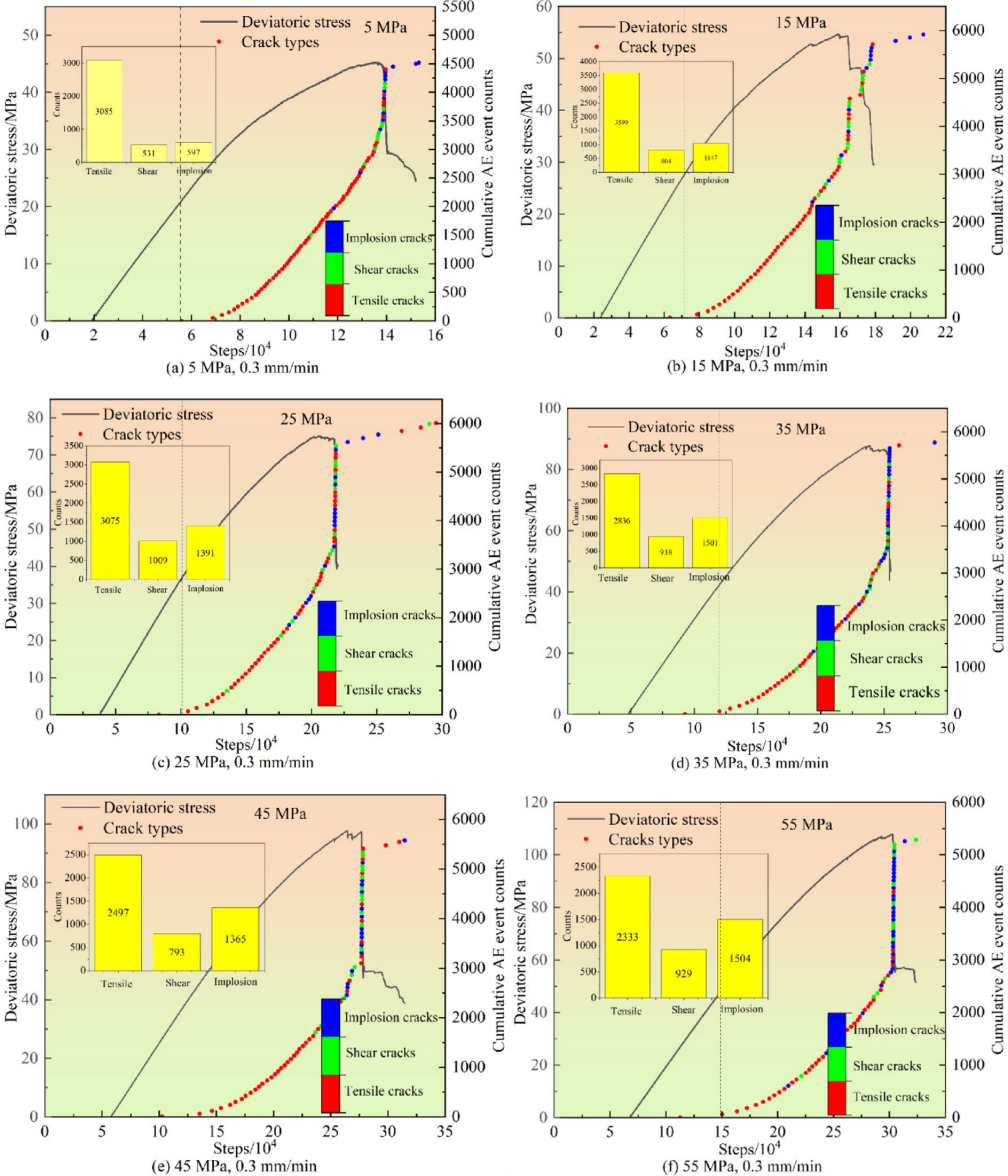
Distribution characteristics of AE events and crack types under different confining pressures.

The distribution characteristics of AE event counts and crack types under different confining pressures are illustrated in Fig. 8. By calculating crack types through isotropic components, three categories are identified: tensile cracks (red), shear cracks (green), and mixed cracks (blue). The results reveal similar trends in AE behavior and crack propagation across all confining pressures: (1)No AE signals are detected in the initial loading stage due to the exclusion of the compaction phase in the numerical model; (2)Stable crack propagation occurs during the elastic stage with minor AE signals; (3)Accelerated crack growth during the plastic stage leads to a significant increase in AE events, reaching a peak at the ultimate strength; (4)Stable crack growth with limited AE activity dominated by red-marked tensile cracks during the elastic stage; (5)Unstable crack propagation with continuously increasing AE events during the plastic stage.

Although tensile cracks dominated at this stage, shear and mixed cracks emerged with increasing stress. Notably, at the peak stress of the specimen, the AE event count reached its maximum with dense crack clusters, where shear and mixed cracks (green and blue dots in the figure) increased significantly and became dominant. This observation aligns with the experimental results under uniaxial compression.

As shown in Table 3, the proportions of different types of cracks under different confining pressures are presented. It can be observed that when the confining pressures are 5 MPa, 15 MPa, 25 MPa, 35 MPa, 45 MPa, and 55 MPa, the proportions of tensile cracks in the total cracks are 73.2%, 66.0%, 56.1%, 53.7%, 53.25%, and 48.9% respectively. The proportion of tensile cracks gradually decreases as the confining pressure increases. Similarly, as the confining pressure increases from 5 MPa to 55 MPa, the proportion of mixed cracks increases from 14.2 to 31.7%. In other words, the proportion of mixed cracks rises with the increase of the confining pressure.

**Table 3:** Crack proportion under different confining pressure.

Confining pressure/MPa	Tensile crack/%	Shear crack/%	mixed-mode crack/%
5	73.2	12.6	14.2
15	66.0	14.7	19.3
25	56.1	18.4	25.5
35	53.7	17.7	28.6
45	53.25	17.1	29.6
55	48.9	19.4	31.7

#### Determination of crack propagation stages using volumetric strain

This section investigates the relationship between volumetric stiffness and crack propagation stages under triaxial compression conditions. For cylindrical specimens under uniaxial or triaxial loading, the volumetric strain can be expressed as:

5 $$\:{\epsilon\:}_{V}={\epsilon\:}_{1}+2{\epsilon\:}_{3}$$.

where *ε*_₁_ is the axial strain and *ε*_₃_ is the lateral strain.

**Fig. 9:**
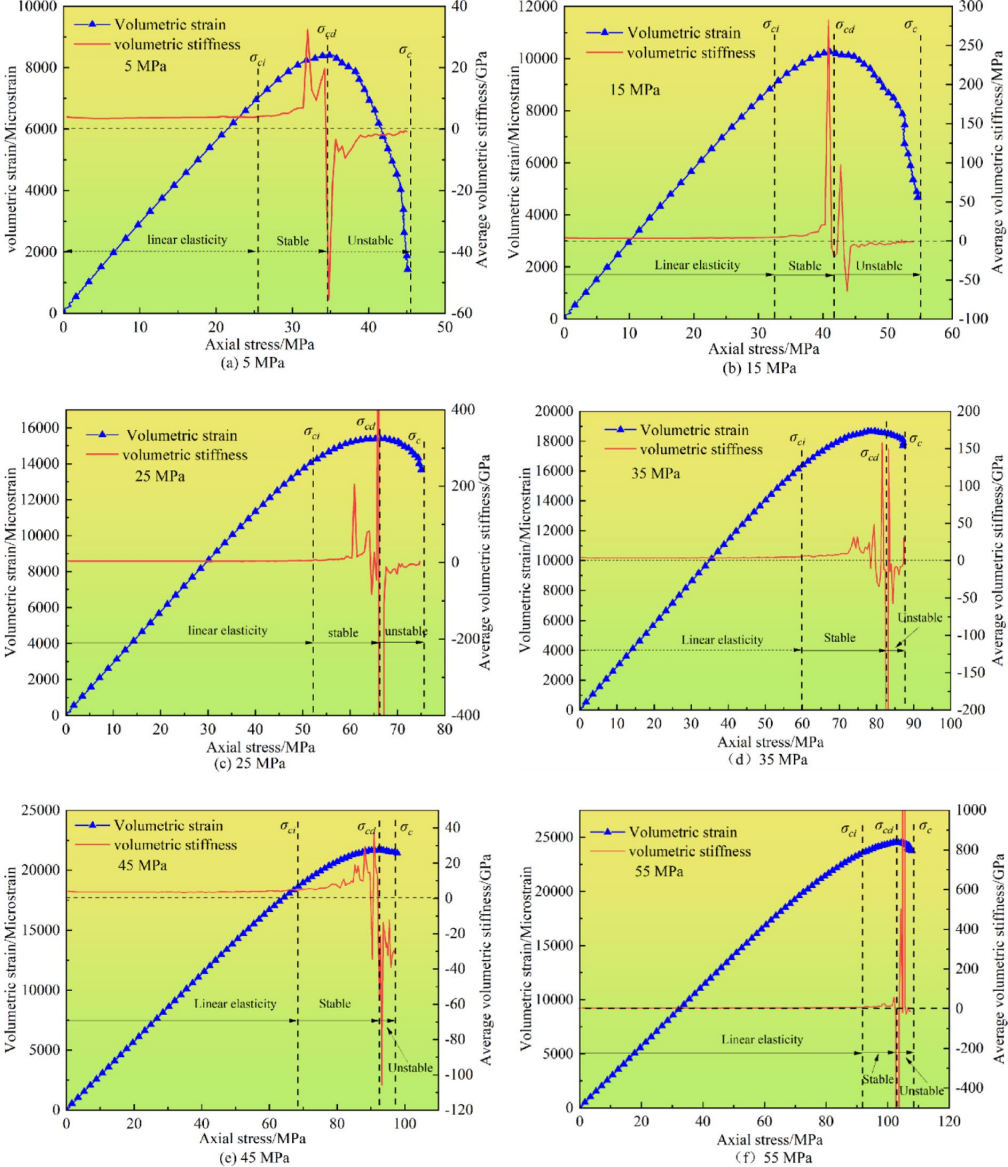
Relationship between volumetric stiffness and failure stage.

Volumetric strain is the combined result of axial and lateral strains. When the specimen is compressed axially, it expands laterally. Assuming that the axial strain gradually increases with stress and is positive, the corresponding lateral strain is negative. As shown in Fig. 9, the volumetric strain is positive. This indicates that under triaxial compression, axial deformation always dominates over lateral deformation throughout the process.

Volumetric strain is closely related to the crack propagation stage. The crack state can be further determined based on the variation trend of volumetric strain. The combination of the moving-point regression method and AE can more accurately determine the crack propagation stage. The volumetric stiffness curve can be regarded as the stress-dependent change rate based on volumetric strain. The volumetric stiffness is calculated as the slope of the volumetric strain versus the axial strain. The change rate of the volumetric strain curve depends on the measured change rates of the axial and lateral strains.

Since it is difficult for the rigid particles in the PFC^2D^ software to simulate the compaction stage of the specimen, the volumetric strain increases linearly at the initial loading stage, which is manifested as a constant straight line on the volumetric stiffness curve. Subsequently, the volumetric stiffness remains as a constant straight line until a certain stress point, indicating that the specimen is in the elastic stage during this period. This stress boundary point is the crack initiation stress threshold σ_*ci*_.

As stress increases, the growth rate of volumetric strain gradually decelerates until reaching its peak value, during which the volumetric stiffness exhibits a slow upward trend. When the lateral strain rate exceeds the axial strain rate, the volumetric strain reverses from positive to negative. These phenomena are clearly reflected in the volumetric stiffness curve: as the volumetric strain approaches the reversal critical point, the abrupt increase in lateral strain rate causes continuous reduction of volumetric strain, leading to an instantaneous transition of volumetric stiffness from positive to negative values. Consequently, the inflection point of volumetric stiffness corresponds to the crack damage stress threshold *σ*_*cd*_. Beyond *σ*_*cd*_, cracks enter an accelerated propagation phase until the specimen reaches peak strength *σ*_*c*_ and fails completely, which corresponds to the unstable crack propagation stage.

Volumetric strain parameters under different confining pressures are presented in Table 4. Combining Fig. 9; Table 4 reveals similar evolutionary trends in volumetric stiffness and volumetric strain across all confining pressures, though the maximum volumetric strain increases progressively with rising confining pressure. During the unstable crack propagation stage, volumetric strain decreases after reversal with reduced amplitude at higher confining pressures. Higher confining pressure delays the initiation of tensile microcracks by restricting lateral deformation, shifting the failure mode from tensile-dominated to shear-dominated. This delays the stress threshold for visible crack initiation (σ_*ci*_), increasing its ratio to peak strength (*σ*_*c*_). For example, at 5 MPa, volumetric strain decreases from 8409 microstrains at damage stress threshold *σ*_*cd*_ to 1428 microstrains at peak stress *σ*_*c*_ (83.01% reduction), while at 55 MPa, it decreases from 24,489 microstrains to 23,737 microstrains (3.07% reduction). This indicates confining pressure suppresses strain variation during unstable crack propagation, enhancing specimen failure resistance. Table 4 further shows that crack initiation stress threshold *σ*_*ci*_, damage stress threshold *σ*_*cd*_, and peak stress *σ*_*c*_ all increase significantly with confining pressure, alongside synchronous increases in *σ*_*ci*_/*σ*_*c*_ and *σ*_*cd*_/*σ*_*c*_ ratios. This suggests confining pressure delays crack nucleation by closing pore structures, leading to earlier crack formation under low confining pressure and reduced damage stage proportion under high confining pressure due to retarded microcrack-to-macrocrack transition.

**Table 4:** Volume strain parameters under different confining pressures.

Confining pressure/MPa	σ_ci_/MPa	σ_cd_/MPa	σ_c_/MPa	σ_ci_/σ_c_	σ_cd_/σ_c_
5	25.62	34.74	45.11	0.56	0.77
15	32.56	41.61	54.67	0.59	0.76
25	52.41	66.12	75.05	0.69	0.88
35	59.86	82.93	87.48	0.69	0.94
45	68.15	92.51	97.46	0.73	0.94
55	92.22	103.12	107.90	0.85	0.95

With the confining pressure increasing from 5 MPa to 55 MPa, the crack types exhibit a marked transition: the proportion of tensile cracks decreases from 73.2 to 48.9%, while shear/mixed-mode cracks gradually become dominant. This microstructural evolution directly governs the macro-response patterns of AE precursor indicators-the tensile-crack-dominated stage corresponds to high-frequency, low-energy AE signals, whereas the increase in shear/mixed-mode cracks tends to generate low-frequency, high-energy signals.

### Coal catastrophe early-warning indicators

The transition of crack types from tensile-dominated to shear/mixed-mode with increasing confining pressure directly links to the evolutionary patterns of AE precursor indicators. This section systematically investigates the dynamic evolutionary patterns of AE early-warning indicators during coal instability under different confining pressures through statistical analysis of AE signals.

#### Definition of AE *b*-value

The *b*-value was originally applied in seismology to describe the relationship between earthquake magnitude and frequency^32^. This parameter helps assess the temporal characteristics of earthquake sequences and analyze potential hazards. Investigating *b*-value holds critical significance for understanding earthquakes and other geological hazards. Its empirical formulation is known as the Gutenberg-Richter relationship^33^, expressed as:

6 $$\:{log}_{10}N(N\ge\:M)=a-bM$$.

Where, *M* represents earthquake magnitude, and *N* denotes the number of earthquakes with magnitudes ≥ *M*. Constants a and b characterize this relationship.

AE signals exhibit distribution characteristics highly similar to earthquake signals, with their amplitudes and frequencies also adhering to the Gutenberg-Richter law^34^. The relationship between AE amplitude (dB) and earthquake magnitude is given by^35^:

7 $$\:{log}_{10}N(N\ge\:M)=a-b\frac{{A}_{dB}}{20}$$.

According to the above formula, the *b*-value relates to the frequency of seismic or AE events across different magnitude ranges. A larger *b*-value indicates relatively higher frequencies of smaller-magnitude earthquakes, whereas a smaller *b*-value may signify higher frequencies of larger-magnitude earthquakes. Additionally, based on the relationship between magnitude and AE signals, a smaller *b*-value corresponds to the generation of high-amplitude AE signals, while a larger *b*-value indicates more frequent low-amplitude AE signals. AE phenomena originate from the elastic wave energy released during crack propagation in coal-rock materials. Therefore, quantitative analysis of *b*-value enables assessment of crack propagation processes in coal masses and prediction of potential catastrophic instability.

#### Definition of AE *β*_*t*_-value

The energy release of AE follows the law of temporal effects^36^:

8 $$\:{E}_{AE}\left(t\right)\propto\:H\left(t\right)\propto\:{t}^{{\beta\:}_{t}}$$.

Where *t* denotes the testing time, *H* represents the cumulative number of monitored AE events, and *β*_*t*_ signifies the scaling exponent of temporal evolution during the damage process.

The degree of material damage and failure can be represented by a function of AE event counts and time:

9 $$\:log\left(\frac{H}{{H}_{d}}\right)={\beta\:}_{t}log\left(\frac{t}{{t}_{d}}\right)$$.

Where *H*_*d*_ denotes the cumulative AE counts within the selected time interval, and *t*_*d*_ represents the monitoring duration.

Clearly, the *β*_*t*_-value can be calculated using the ratio of AE events to total events and the corresponding ratio of time intervals to total time. By linearly fitting log(*H*/*H*_*d*_) against log(*t*/*t*_*d*_), the slope of the resulting line yields the AE *β*_*t*_-value.

#### The effect of confining pressure on *b*-value

**Fig. 10:**
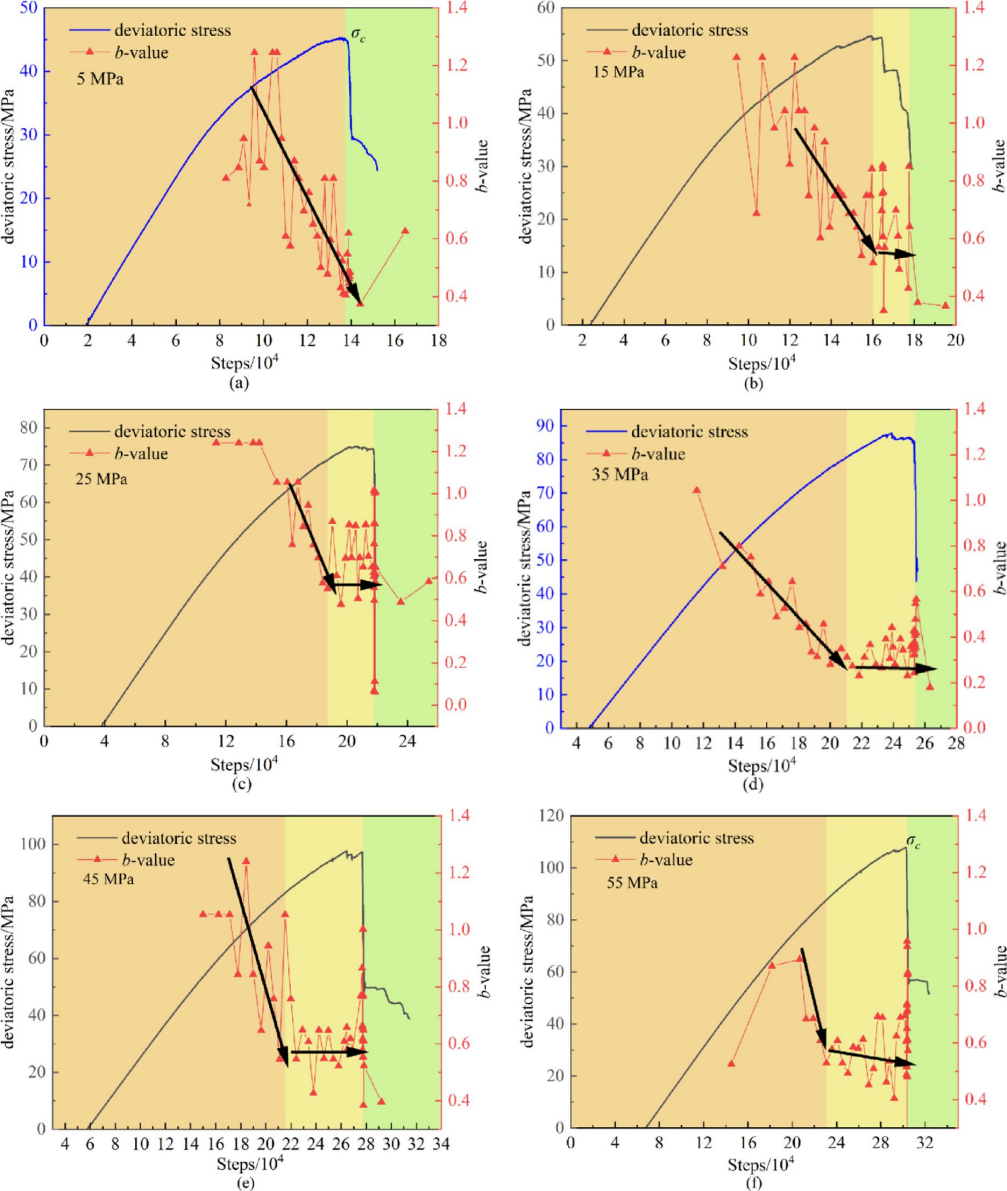
Change curve of b value under different confining pressure.

Consistent with the laboratory AE testing method, the calculation of *b*-value in numerical simulation also employs the time window technique. By calculating the *b*-value for each time window and connecting them sequentially, a curve depicting the variation of *b*-value with time steps is obtained (Fig. 10). Due to the absence of a compaction stage in the numerical model and the low number of AE events during the elastic phase, no significant *b*-value fluctuations were observed in the early loading stage. The initial *b*-value remained stable, attributed to the sparse and low-energy nature of AE events during elastic deformation. As the specimen entered the crack propagation stage, the *b*-value gradually decreased with the continuous initiation and propagation of microcracks. Notably, in the stage approaching failure, the *b*-value exhibited oscillatory downward trends, reaching its minimum at the moment of failure. This phenomenon indicates an alternating development of cracks with varying scales during the crack propagation stage, where unstable crack growth leads to frequent high-energy events dominated by macroscopic rupture. Therefore, the continuous decrease of *b*-value can serve as a precursor for the instability failure of specimens.

Significant differences exist in the evolutionary characteristics of *b*-value under varying confining pressures. Subject to confining pressure constraints, the *b*-value generally exhibits a fluctuating downward trend and remains at a low level prior to failure. Under low confining pressure conditions (Fig. 10(a)), when the confining pressure is 5 MPa, the weak constraint leads to rapid crack propagation, forming high-energy through-cracks within a short period, resulting in a rapid decrease in *b*-value. As the confining pressure increases, however, the compaction effect on pre-existing fractures intensifies while inhibiting the initiation of new cracks, thereby slowing crack development. Once cracks form within the specimen, they rapidly coalesce and propagate, causing an abrupt drop in *b*-value. Due to the confining pressure constraint, most crack propagation remains restricted, and the specimen retains residual load-bearing capacity. At this stage, AE events continue to increase, leading to an initial rapid decrease in *b*-value followed by a stable low-level plateau before failure (Fig. 10(b-f)). This plateau represents a phase of stable shear crack propagation where microcracks coalesce gradually without abrupt energy release. Under high confinement, the dominance of shear/mixed-mode cracks leads to continuous frictional sliding and particle rearrangement, generating a steady stream of moderate-to-high-energy AE events. The low *b*-value signifies a higher proportion of large-magnitude events, reflecting the progressive formation of a dominant shear band. However, the “stability” of the plateau indicates that these microcracks remain spatially distributed and have not yet formed a through-going failure surface.

Specifically, at confining pressures of 15 MPa, 25 MPa, 35 MPa, 45 MPa, and 55 MPa, the durations of low *b*-value prior to failure are approximately 2 × 10^4^, 3 × 10^4^, 5 × 10^4^, 6 × 10^4^, and 7 × 10^4^ steps respectively. This indicates that higher confining pressures prolong the time required for specimen failure and correspondingly increase the duration of low *b*-value stability before failure.

#### The effect of confining pressure on *b*-value

**Fig. 11:**
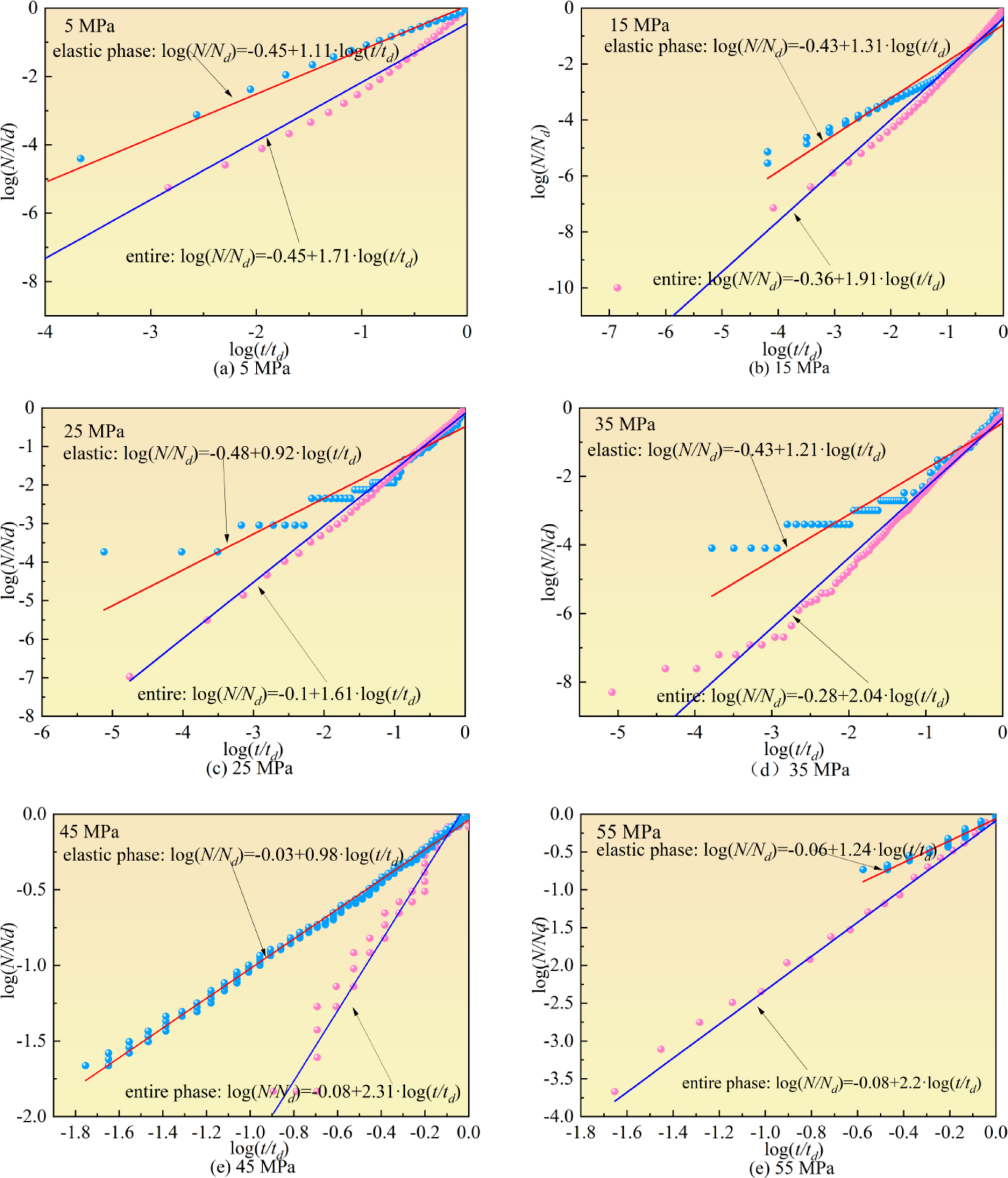
*β*_*t*_-value changes under different confining pressures.

This section systematically analyzes the distribution patterns of *β*_*t*_-value under different confining pressures. The variation characteristics of *β*_*t*_-value at different stress stages under varying confining pressures are illustrated in Fig. 11. The scatter points in the figure represent the relationship between simulated AE event counts and time scales (steps), with the slope of the double logarithmic curve defining the *β*_*t*_-value. In this study, *β*_*t*_-value were calculated by dividing AE events into three stages: elastic phase, crack propagation phase (including stable and unstable propagation), and the entire loading process, following the criteria specified in Fig. 9.

Key findings indicate that Key findings indicate that *β*_*t*_-value during the elastic phase are relatively small, whereas those for the entire loading stage are significantly larger. This discrepancy arises because the sparse AE events during the elastic phase lead to a steady cumulative event count, resulting in synchronous linear growth between events and time. Under confining pressures of 5 MPa, 15 MPa, 25 MPa, 35 MPa, 45 MPa, and 55 MPa, *β*_*t*_-value in the elastic phase are 1.11, 1.31, 0.92, 1.21, 0.98, and 1.24 respectively (all close to 1), while *β*_*t*_-value for the entire loading stage are 1.71, 1.91, 1.61, 1.21, 2.31, and 2.2 respectively (all significantly greater than 1). This phenomenon reflects an acceleration in AE event growth: slow initial growth dominated by microcracks transitions to exponential growth driven by macroscopic crack coalescence near failure, resulting in *β*_*t*_ > 1 characteristics. Therefore, *β*_*t*_-value less than 1 indicate stable specimen deformation, while *β*_*t*_-value greater than 1 serve as a precursor criterion for entering an unstable state.

Further analysis reveals no significant influence of confining pressure on *β*_*t*_-value evolution. *β*_*t*_-value in the elastic phase remain close to 1 across all confining pressures, and although *β*_*t*_-value for the entire loading stage are consistently > 1, no systematic trends with increasing confining pressure are observed. This stems from the fact that *β*_*t*_-value fundamentally depend on the selected time scale, and confining pressure merely alters crack propagation rates without significantly modifying the power-law relationship between AE events and time. Thus, this study concludes that *β*_*t*_-value evolution exhibits no direct correlation with confining pressure magnitude.

## Conclusions

This study employs the PFC^2D^ discrete element numerical simulation software to conduct numerical simulation tests on cylindrical specimens under triaxial conditions, analyzing the strength and deformation characteristics of specimens under different confining pressures. Based on moment tensor analysis, the AE during specimen compressive failure was simulated, and the AE response characteristics under different confining pressures were analyzed. The crack propagation laws and evolutionary patterns of early-warning indicators were also discussed. The main conclusions are as follows:

1. Peak strength increases progressively with increasing loading rates. Under low confining pressures, peak strength demonstrates high sensitivity to loading rate variations, whereas under higher confining pressures, peak strength remains relatively unchanged with increasing loading rates.
2. AE curves under different confining pressures show similar trends. During the elastic stage, AE events remain sparse and dominated by tensile cracks. In the unstable crack propagation stage, shear cracks and mixed-mode cracks continuously emerge and become dominant. With increasing confining pressure, the proportion of tensile cracks gradually decreases while the proportions of shear and mixed-mode cracks increase.
3. The variation rate of volumetric strain with respect to stress can be used to determine crack initiation and damage thresholds. Stress curves can be divided into elastic, stable crack propagation, and unstable crack propagation stages based on the trends of volumetric stiffness. As confining pressure increases, both crack initiation and damage stress thresholds increase, with the proportion of initiation stress increasing while the duration of the unstable crack propagation stage decreases.
4. Under confining pressure, microcracks develop confined, allowing residual load-bearing capacity. AE events drive b-value to exhibit a rapid decline-stable plateau pattern. The acceleration coefficient *βₜ* serves as a universal instability indicator: *βₜ*<1 signals stable growth, *βₜ*>1 indicates instability, with *βₜ* independent of confining pressure. Unlike traditional *b*-value, this pressure-insensitive *βₜ* enables robust early warning across diverse conditions.

## Data Availability

The datasets used and/or analysed during the current study available from the corresponding author on reasonable request.

## Abbreviations

σ_t_: Tensile strength
c: Cohesion
E: Elastic modulus
σ_c_: Uniaxial compressive strength
ε_m_: Maximum axial strain
ε_c_: Critical strain at damage threshold
φ: The angle of internal friction
R_min_: Minimum particle size
R_max_: Maximum particle size
n: Porosity
